# Ideal vitamin D and handgrip strength counteracts the risk effect of APOE genotype on dementia: a population-based longitudinal study

**DOI:** 10.1186/s12967-023-04195-3

**Published:** 2023-05-29

**Authors:** Jiangtao Feng, Qi Wang, Yuan Zhang

**Affiliations:** 1 Department of Orthopedics, Tianjin NanKai Hospital, Changjiang Road 6, Tianjin, 300100 China; 2 Department of Orthopedics, Integrated Chinese and Western Medicine Hospital, Tianjin University, Changjiang Road 6, Tianjin, 300100 China; 3 Raymond G. Perelman Center for Cellular and Molecular Therapeutics, Children’s Hospital of Philadelphia, Philadelphia, PA 19104 USA; 4 School of Public Health, Tianjin Medical University, Tianjin, 300070 China

**Keywords:** Vitamin D, Grip strength, APOE e4 genotype, Dementia, Prospective cohort study

## Abstract

**Background:**

Higher vitamin D concentrations and grip strength contribute to lower individual-level risk of dementia, while apolipoprotein 4 (APOE e4) genotype carries increases dementia risk, but whether combination of ideal vitamin D and grip strength counteracts the risk effect of dementia related to APOE e4 genotype remains unclear. We aimed to investigate the interactions between vitamin D/grip strength and APOE e4 genotype and their association with dementia.

**Methods:**

The UK Biobank cohort comprised 165,688 dementia-free participants (aged at least 60 years) for the dementia analysis. Dementia was ascertained using hospital inpatient, mortality, and self-reported data until 2021. Vitamin D and grip strength were collected at baseline and divided into tertiles. APOE genotype was coded as APOE e4 non-carries and APOE e4 carries. Data were analyzed using Cox proportional hazard models and restricted cubic regression splines, with adjusted for known confounders.

**Results:**

Over the follow-up (median: 12.0 years), 3917 participants developed dementia. In women and men, respectively, compared with to the lowest tertile of vitamin D, the HRs (95% CIs) of dementia were lower in the middle [0.86 (0.76–0.97)/0.80 (0.72–0.90)] and the highest tertile [0.81 (0.72–0.90)/0.73 (0.66–0.81)]. Tertiles of grip strength showed similar patterns. In women and men, respectively, participants who had both highest tertile of vitamin D and grip strength was associated with a lower risk of dementia compared to those with both lowest tertile of these two exposures among APOE e4 genotype carries (HR = 0.56, 95% CI 0.42–0.76, and HR = 0.48, 95% CI 0.36–0.64) and APOE e4 genotype non-carries (HR = 0.56, 95% CI 0.38–0.81, and HR = 0.34, 95% CI 0.24–0.47). There were significant additive interactions between lower vitamin D/grip strength and APOE e4 genotype on dementia among women and men.

**Conclusions:**

Higher vitamin D and grip strength were associated with a lower risk of dementia, and seemed to halve the adverse effects of APOE e4 genotype on dementia. Our findings suggested that vitamin D and grip strength may be imperative for estimating the risks of dementia, especially among APOE e4 genotype carries.

**Supplementary Information:**

The online version contains supplementary material available at 10.1186/s12967-023-04195-3.

## Background

Dementia—a group of symptoms affecting memory, thinking, behavior and social abilities severely enough to interfere with person’s activities of daily living and social autonomy [1]. The common form of dementia included Alzheimer’s disease and vascular dementia [2]. Dementia is currently the seventh leading cause of death among all diseases and one of the major causes of disability and dependency among older people globally [3]. Currently, there are more than 55 million people live with dementia worldwide, and nearly 10 million new cases every year [4]. With life expectancy on the rise throughout the world and the proportion of older people in the population is increasing, this number is expected to increase to 78 million in 2030 and 139 million in 2050 [4]. There is currently no treatment available to cure dementia [5]. Anti-dementia medicines and disease-modifying therapies developed to date have limited efficacy, though numerous new treatments are being investigated in various stages of clinical trials. Therefore, identifying the preventable risk factors for dementia is of high priority.

Evidence indicates that serum 25-hydroxyvitamin D, the main biomarker of vitamin D status, may impact brain health because of its neuroprotective, anti-inflammatory, and antioxidant properties [6–8]. Several previous studies have examined the link between vitamin D and cognitive function or dementia, but findings have been inconsistent-protective and no association were equally reported [7, 9]. In a longitudinal study of 1759 non-demented older (≥ 65 years) participants, Chen et al. reported that higher vitamin D intake was associated with a decreased risk of dementia [10]. While the Canadian Study of Health and Aging conducted by Caroline et al. suggested that no significant association was found between vitamin D and cognitive decline, dementia, or Alzheimer’s disease [7]. Handgrip strength weakness, the age-related decline in muscle strength and functional capability, has been found as a risk factor for dementia. Recently, a randomized controlled trial was performed to evaluate the effect of longitudinal supplementation of vitamin D on muscle strength in adult twins, and found that muscle strength in left hand grip increased 18% in participants who received the supplement [11], which suggested that vitamin D and handgrip strength may jointly impact the incidence of dementia. But there has been no investigation of the joint effects, which may be important to provide additive potential benefits in preventing the risk of dementia.

Dementia is multifactorial disease that results from the complex interplay between genetic factors and environmental exposures. Dementia is of highly heritable [2, 12, 13], and emerging evidence demonstrated that apolipoprotein 4 (APOE e4) genotype is the most common genetic risk factor for dementia [2, 14]. A growing amount of epidemiological evidence suggests that several lifestyle factors (e.g., smoking, physical activity, and fish intake) and environmental factors (e.g., sunlight, greenspace) may interact with APOE alleles to synergistically affect the risk of dementia development [15–18]. However, wheather vitamin D and grip strength may jointly modify the effect of APOE genotype on dementia remain largely unknown.

In the present study, we aimed to examine the separate and combination of serum vitamin D and grip strength and the risk of incident dementia, and to investigate whether such associations were modified by genetic predisposition among women and men.

## Methods

### Study design and population

**Fig. 1:**
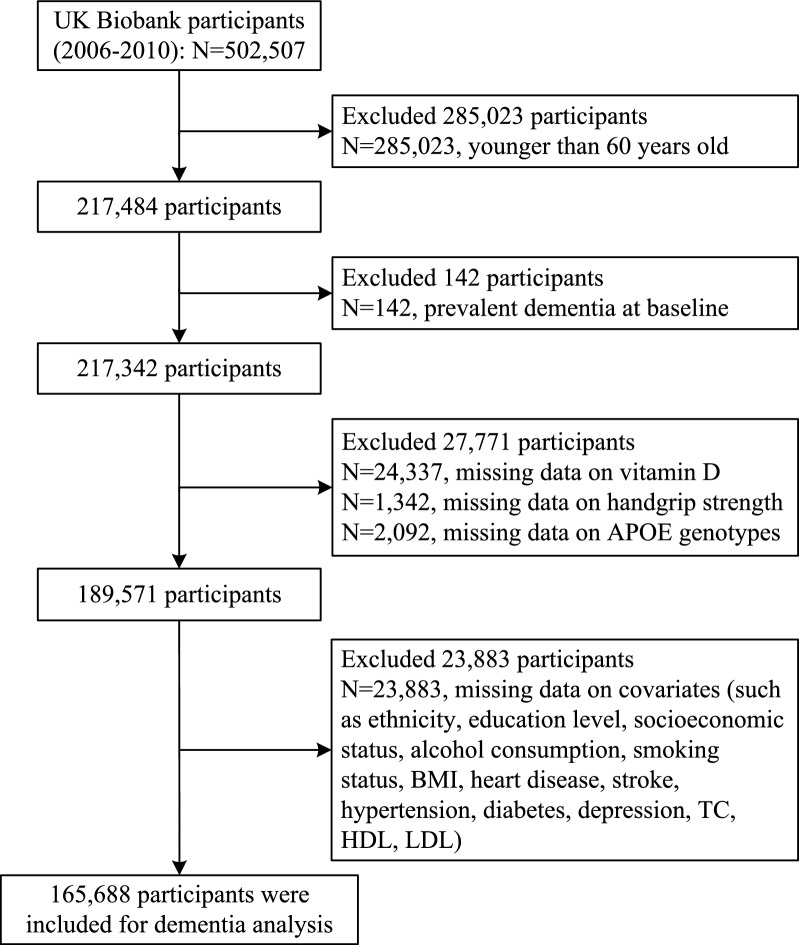
Follow chart for the selection of samples from UK Biobank. APOE, apolipoprotein E; BMI, body mass index; HDL, high density lipoprotein; LDL, low density lipoprotein; TC, cholesterol

The data were derived from UK Biobank, a population-based cohort that recruited 502,412 participants aged 37–73 years who attended one of 22 assessment centers across the United Kingdom between 2006–2010 and were followed up until 2021 [19]. Participants provided completed touch-screen questionnaires, physical examination, and biological samples. We excluded participants with prevalent dementia or less than 60 years old or missing information on genetic factors, vitamin D, and grip strength. Data from 165,688 participants were available for analyses in the present study (Fig. 1). All the UK Biobank participants gave written informed consent before data collection.

### Covariates

Covariates were documented, including age, ethnicity (White, Black, Asian, and other), education level (upper secondary, lower secondary, vocational, and other), socioeconomic status (defined based on the Townsend deprivation index, encompassing information on social class, employment, car availability, and housing), alcohol consumption (never, former, and current), smoking status (never, former, and current), physical activity (active and inactive), diet (healthy and unhealthy), BMI (< 25 kg/m^2^, 25 to 30 kg/m^2^, and ≥ 30 kg/m^2^), heart disease (no, yes), stroke (no, yes), hypertension (no, yes), diabetes (no, yes), depression (no, yes; including depression symptoms, self-reported depression, and inpatient depression), cholesterol, high density lipoprotein cholesterol (HDL), and low density lipoprotein cholesterol (LDL). Regular physical activity was defined as engaging in moderate activity ≥ 150 min/week, vigorous activity ≥ 75 min/week, or moderate and vigorous activity ≥ 150 min/week [20]. A healthy diet was based on adequate intake of at least three of these five commonly eaten food groups (Vegetables ≥ 3 servings/day; Fruits ≥ 3 servings/day; Unprocessed red meats ≤ 1.5 servings/week; Processed meats ≤ 1 serving/week; Fish ≥ 2 servings/week) [21]. Depression symptoms were measured by 4 items from the Patient Health Questionnaire [22]: depressed mood, unenthusiasm/disinterest, tenseness/restlessness, and tiredness/lethargy.

### Assessment of dementia

All-cause dementia was ascertained based on a self-reported diagnosis of dementia (Data-Field 20,002, code: 1263), hospital records (ICD-10 codes: F00, F01, F02, F03, F05.1, G30, G31.1, G31.8), and death records (ICD-10 codes: F00, F01, F02, F03, F05.1, G30, G31.1, G31.8) from the Hospital Episode Statistics (England), the Scottish Morbidity Record (Scotland), and the Patient Episode Database (Wales).

### APOE genotyping

Two single nucleotide polymorphism loci (SNPs), rs429358 and rs7412, determining the possible APOE isoforms, and forming three haplotypes (e2, e3, and e4) and six genotypes (e2/e2, e2/e3, e2/e4, e3/e3, e3/e4, e4/e4). APOE genotype was coded as APOE e4 non-carries (APOE e4−, low genetic risk) and APOE e4 carries (APOE e4+, high genetic risk).

### Assessment of serum 25(OH)D concentrations

Information on biochemistry makers were obtained from biological samples collected at study recruitment (2006–2010) [23, 24]. Serum 25(OH)D concentrations, a measure of vitamin D status, was measured by chemiluminescence technology analysis on a DiaSorin Ltd. LIASON XL [25]. Vitamin D was imputed with the minimum detectable value (10 nmol/L) if it was below the limit of detection [26], and the maximum detectable value (125 nmol/L) if too high for detection (< 0.1%). Vitamin D was categorized into three groups (low, middle, and high) according to tertiles.

### Assessment of grip strength

Grip strength was measured using a Jamar J00105 hydraulic hand dynamometer. Isometric grip force was assessed from a single 3-s maximal grip effort of the right- and left-side arms with participants seated upright with their elbow by their side and flexed at 90° so that their forearm was facing forward and resting on an armrest. The mean of the right and left values was expressed in absolute units (kilograms), as reported elsewhere and was used in the current study. Due to biological differences in grip strength within sex, we divided participants into three groups according to sex-specific categories as low (the lowest tertile), middle (the middle tertile), and high (the highest tertile).

### Statistical analyses

We calculated incidence rates and 95% confidence intervals (95% CIs) per 1000 person-years for dementia among women and men. Multivariable restricted cubic regression splines were used to visual evaluate the relationship between vitamin D, grip strength and incidence of dementia, with four knots at the 25th, 50th, 75th, and 95th centiles. The *P* for overall model < 0.05 and *P* for non-linear < 0.05 indicated a non-linear relationship between vitamin D or grip strength and dementia, while *P* for overall model < 0.05 and *P* for non-linear > 0.05 showed a linear relationship. The cubic regression splines models were adjusted for age, ethnicity, education level, socioeconomic status, alcohol consumption, smoking status, BMI, heart disease, stroke, hypertension, diabetes, depression, TC, HDL, LDL, APOE e4 genotype, and adjusted for vitamin D in grip strength analysis or for grip strength in vitamin D analysis. Association of vitamin D or grip strength categories (tertiles 1 as reference) and prospective dementia incidence were investigated by using Cox proportional hazards regression models with follow-up year as the time scale. Hazard ratios (HRs) and 95% confidence interval (CIs) were used to reported the results. Follow-up time was calculated as the time from baseline assessment until the first event of dementia, death, or March 31, 2021, whichever occurred first. Schoenfeld residuals method was used to check the proportional hazards assumptions. The Kaplan–Meier survival curve were applied to assess the association between joint exposure of vitamin D and grip strength categories and APOE e4 genotype with risk of incident dementia. To examine whether APOE e4 genotype modified the associations of vitamin D and/or grip strength with the risk of dementia, additive and multiplicative interactions were assessed by fitting the relevant parameters into the models. All models were adjusted for age, ethnicity, education level, socioeconomic status, alcohol consumption, smoking status, BMI, heart disease, stroke, hypertension, diabetes, depression, TC, HDL, LDL, APOE e4 genotype, and adjusted for vitamin D in grip strength analysis or for grip strength in vitamin D analysis. If data were missing for a covariate, we used multiple imputations based on five replications and utilized a chained-equation method to account for the missing data [27].

Several additional analyses were performed to assess the robustness of our study results. First, we used stratification analysis to examine whether the association between dementia and the combined association of vitamin D/grip strength and APOE e4 genotype varied by age (< 60 vs. ≥ 60 years), ethnic background, education level, and socioeconomic status. Second, to address the role of potential reverse causality, we repeated the main analyses by excluding participants who developed dementia in the first 3-year follow-up period and participants who died within 3 years from baseline. Furthermore, we excluded participants with major chronic disease, such as heart disease, stroke, diabetes, or cancer at baseline to assess the robustness of our study results.

All analyses were performed using STATA 15 statistical software (Stata Corp, College Station, TX, USA) and R (version 3.6.1, R Foundation for Statistical Computing). All *P*-values were two-sided, and statistical significance was set at 0.05.

## Results

### Baseline characteristics of the study population

Of the 165,688 dementia-free participants, the mean age was 64.1 ± 2.9 years, and 81,078 (48.9%) were men. Over the follow-up (median: 12.0 years, IQR: 11.3 to 12.6), 3917 participants developed dementia. The baseline characteristics of the participants by incident dementia status among women and men are provided in Table 1. In women and men, respectively, participants who developed dementia were more likely to be older, excessive alcohol consumption, former or current smokers, obesity, APOE e4 carriers, had low education attainment and socioeconomic status, had high prevalence of heart disease, stroke, hypertension, diabetes, and depression, and less likely to have higher level of vitamin D and handgrip strength comparted to those who did not develop dementia. Additionally, there was a correlation between vitamin D and handgrip strength in both women (r = 0.011, P = 0.0018) and men (r = 0.051, P < 0.001). The scatter plot of the relationship between handgrip strength and vitamin D was shown in Additional file 1: Figure S1.

**Table 1:** Participant characteristics at baseline by sex and incident dementia status in the UK Biobank

Characteristic	Women		Men	
	All women	Women who developed dementia	All men	Men who developed dementia
Number of participants, n (%)	84,610 (97.9)	1778 (2.1)	81,078 (97.4)	2139 (2.6)
Age, mean (SD), year	64.0 (2.8)	65.8 (2.7)	64.2 (2.9)	65.8 (2.7)
Ethnicity, n (%)				
White	82,300 (97.3)	1723 (96.9)	78,890 (97.3)	2057 (96.2)
Asian	264 (0.3)	5 (0.3)	204 (0.3)	8 (0.4)
Black	780 (0.9)	16 (0.9)	1036 (1.3)	33 (1.5)
Other	1266 (1.5)	34 (1.9)	948 (1.1)	41 (1.9)
Education level, n (%)				
College or University	19,949 (23.6)	303 (17.0)	23,716 (29.3)	493 (23.0)
Upper secondary	7911 (9.3)	151 (8.5)	6990 (8.6)	174 (8.1)
Lower secondary	22,752 (26.9)	431 (24.2)	15,854 (19.6)	360 (16.8)
Vocational	3635 (4.3)	66 (3.7)	8568 (10.6)	242 (11.3)
Other	30,363 (35.9)	827 (46.5)	25,950 (32.0)	870 (40.7)
Socioeconomic status, n (%)				
High	30,104 (35.6)	534 (30.0)	29,933 (36.9)	700 (32.7)
Middle	29,499 (34.9)	564 (31.7)	27,667 (34.1)	715 (33.4)
Low	25,007 (29.6)	680 (38.2)	23,478 (29.0)	724 (33.8)
Moderate alcohol consumption, n (%)	45,434 (53.7)	842 (47.4)	48,915 (60.3)	1,224 (57.2)
Smoking status, n (%)				
Never	48,461 (57.3)	938 (52.8)	33,959 (41.9)	798 (37.3)
Former	30,537 (36.1)	687 (38.6)	39,164 (48.3)	1114 (52.1)
Current	5612 (6.6)	153 (8.6)	7955 (9.8)	227 (10.6)
Grip strength, mean (SD), nmol/L	21.3 (5.7)	19.7 (5.8)	37.2 (8.1)	34.3 (8.2)
Grip strength, n (%)				
Low	37,425 (44.2)	990 (55.7)	33,886 (41.8)	1187 (55.5)
Middle	31,432 (37.1)	570 (32.1)	29,770 (36.7)	676 (31.6)
High	15,753 (18.6)	218 (12.3)	17,422 (21.5)	276 (12.9)
BMI, mean (SD), kg/m^2^	27.3 (4.9)	27.6 (5.4)	27.9 (4.1)	27.9 (4.4)
BMI, n (%)				
Normal weight	29,567 (34.9)	609 (34.3)	18,873 (23.3)	545 (25.5)
Overweight	34,020 (40.2)	688 (38.7)	41,381 (51.0)	995 (46.5)
Obesity	21,023 (24.8)	481 (27.1)	20,824 (25.7)	599 (28.0)
Heart disease, n (%)	3136 (3.7)	159 (8.9)	8795 (10.8)	391 (18.3)
Stroke, n (%)	1567 (1.9)	72 (4.0)	2525 (3.1)	157 (7.3)
Hypertension, n (%)	39,194 (46.3)	943 (53.0)	44,875 (55.3)	1270 (59.4)
Diabetes, n (%)	4295 (5.1)	195 (11.0)	7547 (9.3)	377 (17.6)
Depression, n (%)	677 (0.8)	39 (2.2)	517 (0.6)	30 (1.4)
TC, mean (SD), mmol/L	7.3 (9.4)	6.7 (7.4)	6.5 (8.9)	5.9 (7.5)
HDL, mean (SD), mmol/L	1.6 (0.6)	1.6 (0.6)	1.3 (0.5)	1.3 (0.6)
LDL, mean (SD), mmol/L	5.7 (11.4)	5.5 (11.12)	5.1 (10.6)	4.3 (8.8)
Vitamin D, mean (SD), nmol/L	50.5 (20.3)	47.5 (20.5)	51.5 (20.8)	48.8 (21.2)
Vitamin D, n (%)				
Low	27,528 (32.5)	696 (39.1)	25,297 (31.2)	821 (38.4)
Middle	22,374 (26.4)	453 (25.5)	21,163 (26.1)	526 (24.6)
High	34,708 (41.0)	629 (35.4)	34,618 (42.7)	792 (37.0)
AOPE, n (%)				
APOE e4 non-carrier	61,557 (72.8)	746 (42%)	58,364 (72.0)	1020 (47.7)
APOE e4 carrier	23,053 (27.2)	1032 (58.0)	22,714 (28.0)	1119 (52.3)

APOE, apolipoprotein; BMI, body mass index (calculated as weight in kilograms divided by height in meters squared); HDL, high density lipoprotein; LDL, low density lipoprotein; SD, standard deviation; TC, cholesterol

### Association of vitamin D and handgrip strength with incident dementia

**Fig. 2:**
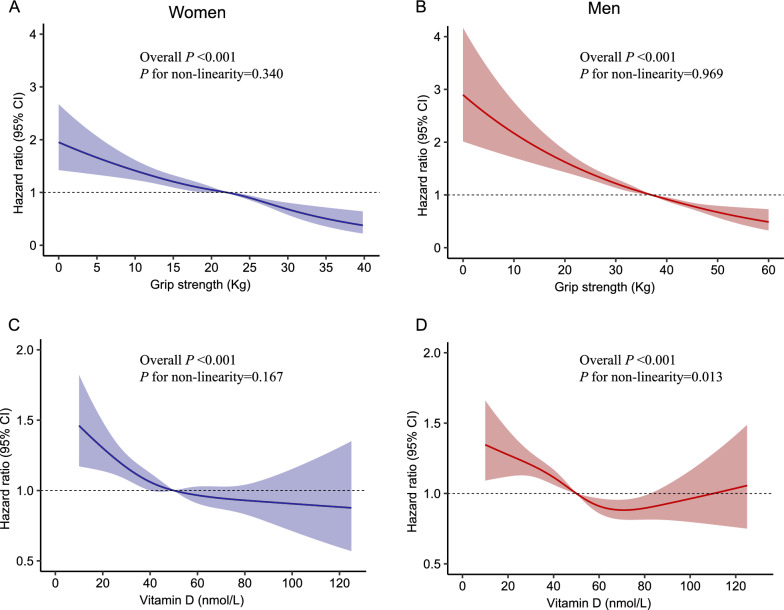
Restricted Cubic Spline models for the relationship between vitamin D and handgrip strength with incident dementia in women and men. **A** Grip strength and dementia in women; **B** Grip strength and dementia in men; **C** Vitamin D and dementia in women; **D** Vitamin D and dementia in men. The 95% CIs of the adjusted hazard ratios are represented by the shaded area. Restricted Cubic Spline model is adjusted for age, ethnicity, education level, socioeconomic status, alcohol consumption, smoking status, BMI, heart disease, stroke, hypertension, diabetes, depression, TC, HDL, LDL, APOE e4 genotype, and we adjusted for vitamin D in handgrip strength analysis or for handgrip strength in vitamin D analysis

Restricted Cubic Spline models were used to evaluate the relationship between vitamin D and handgrip strength with dementia risk. In multi-adjusted models (Fig. 2), handgrip strength was negatively associated with incident dementia. The associations between handgrip strength and dementia were linear in women (*P* for overall model < 0.001 and *P* for non-linear = 0.340) and men (*P* for overall model < 0.001 and *P* for non-linear = 0.969). Per 1-SD increment in handgrip strength was associated with a 3% (HR = 0.97, 95% CI 0.96–0.98) and a 3% (HR = 0.97, 95% CI 0.97–0.98) lower risk of incident dementia among women and men, respectively. In addition, the association between vitamin D and dementia was linear among women (*P* for overall model < 0.001 and *P* for non-linear = 0.167), but the U shape relationship between vitamin D and dementia was found among men (*P* for overall model < 0.001 and *P* for non-linear = 0.013).

When the study categories handgrip strength into three groups according to tertiles, in women and men, respectively, compared with the lowest tertile of handgrip strength, the HRs (95% CIs) of dementia were lower in the middle [0.78 (0.70–0.87)/0.75 (0.68–0.82)] and the highest tertile [0.64 (0.56–0.75)/0.60 (0.52–0.68)]. Tertiles of vitamin D showed a similar pattern. In multi-adjusted Cox models, HRs and 95% CIs of dementia were 0.86 (0.76–0.97)/0.80 (0.72–0.90) for participants with middle vitamin D status and 0.81 (0.72–0.90)/0.73 (0.66–0.81) for those with high vitamin D status, compared to those with low vitamin D status (Table 2).

**Table 2:** Association of handgrip strength and vitamin D with incidence of dementia in women and men

Factors	Cases	Women		Cases	Men	
		Unadjusted HR (95% CI)	Multi-adjusted HR (95% CI)^a^		Unadjusted HR (95% CI)	Multi-adjusted HR (95% CI)^a^
Handgrip strength						
Low	990	1.00 (ref.)	1.00 (ref.)	1187	1.00 (ref.)	1.00 (ref.)
Middle	570	0.67 (0.60–0.74)	0.78 (0.70–0.86)	676	0.64 (0.58–0.70)	0.74 (0.68–0.82)
High	218	0.49 (0.43–0.57)	0.64 (0.56–0.75)	276	0.43 (0.38–0.49)	0.59 (0.51–0.67)
Vitamin D						
Low	696	1.00 (ref.)	1.00 (ref.)	821	1.00 (ref.)	1.00 (ref.)
Middle	453	0.80 (0.71–0.91)	0.86 (0.76–0.97)	526	0.78 (0.70–0.87)	0.79 (0.70–0.88)
High	629	0.72 (0.65–0.80)	0.80 (0.71–0.89)	792	0.72 (0.65–0.80)	0.71 (0.64–0.79)

^a^Multivariate Cox regression models were adjusted for ethnicity, education level, socioeconomic status, alcohol consumption, smoking status, BMI, heart disease, stroke, hypertension, diabetes, depression, TC, HDL, LDL, APOE genotype, and we adjusted for vitamin D in handgrip strength analysis or for handgrip strength in vitamin D analysis

### Joint effect of handgrip strength, vitamin D and APOE e4 genotype on dementia risk

**Fig. 3:**
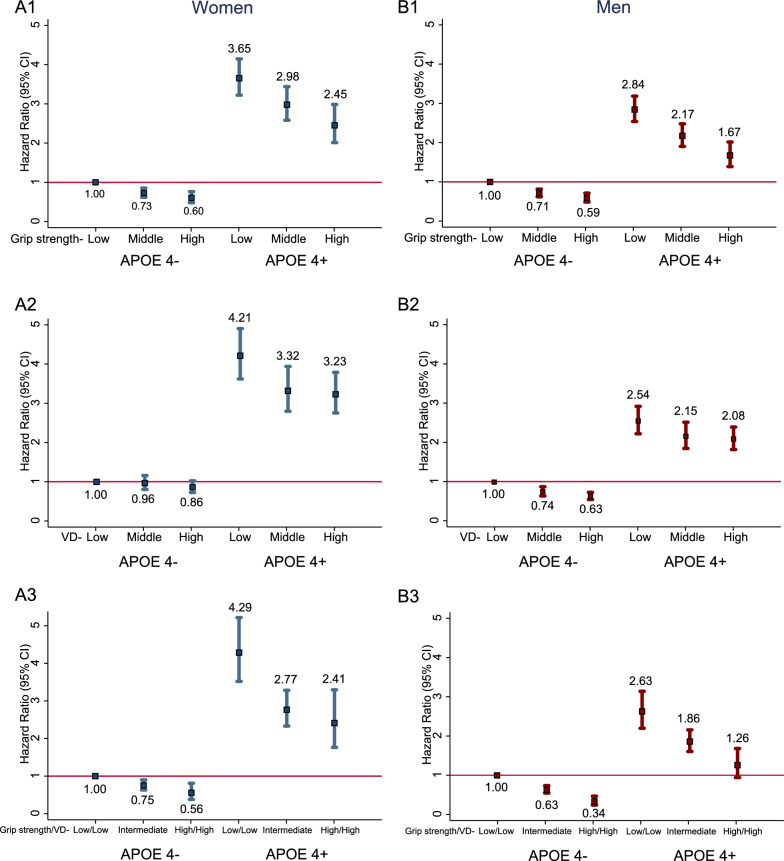
Association of handgrip strength, vitamin D, and APOE e4 genotype with incident dementia risk in women and men. **A1** Joint effect of handgrip strength and APOE e4 genotype on dementia in women; **A2** Joint effect of vitamin D and APOE e4 genotype on dementia in women; **A3** Joint effect of handgrip strength, vitamin D, and APOE e4 genotype on dementia in women; **B1** Joint effect of handgrip strength and APOE e4 genotype on dementia in men; **B2** Joint effect of vitamin D and APOE e4 genotype on dementia in men; **B3** Joint effect of handgrip strength, vitamin D, and APOE e4 genotype on dementia in men. Cox regression model is adjusted for age, ethnicity, education level, socioeconomic status, alcohol consumption, smoking status, BMI, heart disease, stroke, hypertension, diabetes, depression, TC, HDL, LDL, and we adjusted for vitamin D in handgrip strength analysis or for handgrip strength in vitamin D analysis

Figure 3 shows the association between dementia and the joint exposures of handgrip strength, vitamin D, and APOE e4 genotype. After fully-adjusted for confounding factors, compared to participants with APOE e4 non-carries and low handgrip strength, the HRs (95% CIs) of dementia were 3.65 (3.22–4.15) and 2.84 (2.54–3.19) for those with APOE e4 carries plus low handgrip strength among women and men, respectively, and 2.45 (2.01–2.99) and 1.67 (1.39–2.01) for those with APOE e4 carries and high handgrip strength among women and men, respectively. Similar, compared to participants with APOE e4 non-carries and low vitamin D status, the HRs (95% CIs) of dementia were 4.21 (3.62–4.90) and 2.54 (2.22–2.92) for those with APOE e4 carries plus low vitamin D status, and 3.23 (2.75–3.79) and 2.08 (1.82–2.39) for those with APOE e4 carries and high vitamin D status among women and men, respectively.

Furthermore, there was a significant additive interaction between lowest tertile of both handgrip strength and vitamin D and APOE e4 carries that associated with dementia among women (RERI: 2.868, 95% CI 1.048–4.688; AP: 0.352, 95% CI 0.169–0.537; SI: 1.671, 95% CI 1.175–2.375) and men (RERI: 2.020, 95% CI 0.460–3.581; AP: 0.262, 95% CI 0.085–0.439; SI: 1.431, 95% CI 1.069–1.915. In joint effect analysis, compared to participants with both low handgrip strength/low vitamin D and APOE e4 non-carries, the HRs (95% CIs) of dementia were 4.29 (3.52–5.22) and 2.62 (2.2.20–3.14) in those with APOE e4 carries plus low handgrip strength/low vitamin D profiles among women and men, respectively, and 2.41 (1.77–2.33) and 1.26 (0.94–1.68) in those with APOE e4 carries and both high handgrip strength and vitamin D profiles among women and men, respectively. In women and men, respectively, participants who had both high vitamin D and grip strength was associated with lower risk of dementia compared to those with both low vitamin D and grip strength among APOE e4 genotype carries (HR = 0.56, 95% CI 0.42–0.76, and HR = 0.48, 95% CI 0.36–0.64) and APOE e4 genotype non-carries (HR = 0.56, 95% CI 0.38–0.81, and HR = 0.34, 95% CI 0.24–0.47).

**Fig. 4:**
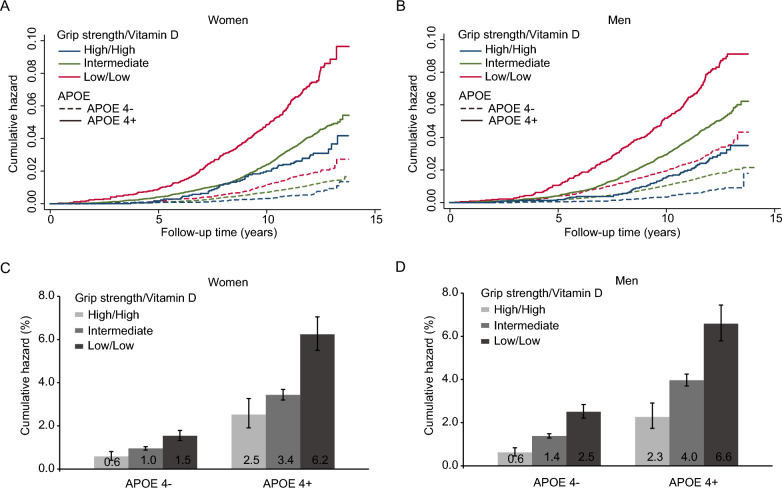
Incidence of dementia by joint effect of handgrip strength, vitamin D, and APOE e4 genotype in women and men. **A** Cumulative incidence of dementia during 15 years of follow-up by joint effect of handgrip strength, vitamin D, and APOE e4 genotype in women; **B** Cumulative incidence of dementia during 15 years of follow-up by joint effect of handgrip strength, vitamin D, and APOE e4 genotype in men; **C** Cumulative incidence of dementia per 1000 person-years at 15 years of follow-up by joint effect of handgrip strength, vitamin D, and APOE e4 genotype in women; **D** Cumulative incidence of dementia per 1000 person-years at 15 years of follow-up by joint effect of handgrip strength, vitamin D, and APOE e4 genotype in women. Error bars represent 95% CI of estimated cumulative incidence

The Kaplan–Meier survival curves showed that the risk of incident dementia was highest for APOE e4 carries with both lowest tertile of handgrip strength and vitamin D profiles among women and men (Fig. 4A, B). Of the APOE e4 carries with both low vitamin D and grip strength, 6.4% (95% CI 5.9–7.0%) incident dementia compared to 2.4% (95% CI 2.1–2.7%) of APOE e4 carries with both high vitamin D and grip strength (Fig. 4C, D).

### Additional analyses

Association between dementia and the combined association of vitamin D/grip strength and APOE ε4 genotype did not meaningfully differ by age (Additional file 1: Table S1), education level (Additional file 1: Table S2), and socioeconomic status (Additional file 1: Table S3; all *P* for interaction > 0.05). The results were not much altered compared with those from initial analyses when we repeated analyses by excluding participants who developed dementia in the first 3-year follow-up period and participants who died within 3 years from baseline (Additional file 1: Table S4), or by excluding participants with major chronic disease, such as heart disease, stroke, diabetes, or cancer at baseline (Additional file 1: Table S5). Additionally, we estimated the association between vitamin D and the incidence of dementia stratified by APOE e4 genotype. In women, participants who had highest tertile of vitamin D was associated with lower risk of dementia compared to those with lowest tertile of vitamin D among APOE e4 carries (HR = 0.73; 95% CI 0.63–0.85), but not among APOE e4 non-carries (HR = 0.91; 95% CI 076–1.08). In men, compared to participants with lowest tertile of vitamin D, those with the highest tertile of vitamin D had a lower risk of dementia in both APOE e4 non-carries (HR = 0.65, 95% CI 0.56–0.75) and APOE e4 carries (HR = 0.80, 95% CI 0.69–0.92) (Additional file 1: Table S6).

## Discussion

We found that participants with higher vitamin D and grip strength profiles were associated with lower risk of dementia, and seemed to counteract the adverse effects of APOE e4 genotype on dementia by almost 50%. Of the APOE e4 carries with both low vitamin D and grip strength, 6.4% incident dementia compared to 2.4% of APOE e4 carries with both high vitamin D and grip strength.

The association of higher vitamin D with lower risk of developing dementia supported several previous studies. Klodian et al. conducted a population-based prospective cohort study over 12 years of follow-up, and found that dietary vitamin D was associated with a slower rate of decline in cognitive function among Blacks [28]. In a longitudinal study of 1759 non-demented older (≥ 65 years) participants, Chen et al. reported that higher vitamin D intake was associated with a decreased risk of dementia [10]. In our study, we found that lower vitamin D levels were associated with reduced risk of dementia, and there was a non-linear relationship in women, but a non-linear relationship in men. Further large-scale population-based prospective studies are needed to verify our findings.

Our findings regarding the association between vitamin D levels and handgrip strength supported previous researches. Kalliokoski et al. found that even moderate intake of supplemental vitamin D and calcium can improve grip strength and upper leg performance in pregnant and recently pregnant women [29]. Ewid et al. also confirmed that standard 25(OH)D oral supplementation improved muscle strength and quality of life in adult Saudi females [30]. Moreover, case–control studies and systematic reviews have reported a positive correlation between vitamin D levels and handgrip strength in various populations, including Indian hip fracture subjects [31], Mexican community-dwelling older women [32], and postmenopausal women [33]. However, our reported correlative coefficients (r = 0.011 for women and r = 0.051 for men) were lower than those reported in Kocak’s study (r = 0.362) and Dhanwal’s study (r = 0.482), possible due to differences in study populations, sample size, as well as ethnicity background. Additionally, people who volunteer for the UK Biobank cohort tend to be, on average, more health-conscious than nonparticipants, which may lead to underestimation the correlation between Vitamin D and grip strength [34].

Many studies have investigated the relationship between vitamin D and APOE genotype, but with inconsistent findings. Some epidemiological studies suggest that higher 25(OH)D concentrations might be particularly beneficial for memory function in individuals with two APOE 4 alleles [35], while another study indicates that vitamin D deficiency present a greater risk for APOE e4 non-carrier Alzheimer’s disease patients than for e4 carriers [36]. The National Alzheimer’s Coordinating Center (NACC) conducted a prospective cohort study that reported exposure to vitamin D was associated with significantly lower incidence of dementia in both APOE ε4 carriers and non-carriers, but the effect was greater in non-carriers [37]. There are also data indicating that APOE e4 carriers have higher 25(OH)D concentrations in targeted replacement mice and humans, which is being interpreted as a potential evolutionary adaptation [38]. Our study also suggested that higher Vitamin D levels might be particularly beneficial for dementia among APOE e4 genotype carries in women, while in men, higher vitamin D levels appeared to benefit both APOE ε4 carriers and non-carriers, but the effect was greater in non-carriers. Our findings in men support the NACC cohort study [37], but differ from previous findings that APOE e4 carriers have higher 25(OH)D concentrations [38], and the cause of this difference might be our prospective cohort study design, the varied ethnicity background and population characteristics. Additionally, the UK Biobank participants were volunteers who were not randomly selected and generally live in less socioeconomically deprived areas. Compared to the general population, they have better health conditions [34], which may cause our findings differ from the aforementioned research [38]. Further clinical studies are needed to verify our findings.

Both handgrip strength and vitamin D were significantly associated with the reduced risk of dementia and dementia-related risk factors, such as diabetes, stroke, or depression [39, 40]. However, whether the combination of the two exposures may additively mitigate the risk of dementia remains largely unknown. Our findings suggested that combination of high vitamin D and handgrip strength counteracts the risk effect of APOE e4 genotype on dementia by almost 50%. There is currently no treatment available to cure dementia. Our study may provide an important perspective for the early prevention and intervention of dementia, especially for genetically susceptible individuals (APOE genotype carries) who are recommended to improve handgrip strength and avoid vitamin D deficiency to reduce the risk of dementia related genetic risk.

The potential mechanisms underlying the interaction between handgrip strength, vitamin D, and APOE genotype remains unclear, and the explain may be that both optimal handgrip strength and vitamin D were associated with lower risk of dementia-related risk factors, such as stroke, diabetes, and depression [39, 41], while studies have confirmed significantly interaction between the aforementioned risk factors and APOE genotype in relation to dementia or cognitive function [42, 43]. Therefore, the two optimal exposures may jointly interact with dementia related genetic risk. Emerging evidence confirmed that a higher frequency of participation in physical activity can improve handgrip strength and increase vitamin D level, and the interaction between physical and APOE genotype have been reported [44, 45]. Brain structural alteration may be another potential mechanism. Three prospective waves from Healthy Aging in Neighborhoods of Diversity Across the Life Span study suggested that serum 25(OH)D status and increase were consistently linked to larger occipital and parietal white matter integrity volumes and greater region-specific white matter integrity [46]. Nevertheless, evidence from a British birth cohort found that lower grip strength from midlife was associated with smaller whole-brain volume and higher white matter hyperintensity volume [47]. Further population-based prospective studies are needed to verify our findings.

The strengths of our study including its large-scale samples, the population-based prospective design with a long follow-up time. This study also has several limitations. First, vitamin D and handgrip strength were measured once at baseline, and potential changes during follow-up may have affected our risk estimates. Second, UK Biobank participants tend to be more health-conscious than nonparticipants, which might lead to underestimating prevalence and incidence of dementia [34]. Third, there remains unmeasured confounding factors similar to most observational studies. Additionally, there appears to be a direct causative relationship between vitamin D and handgrip strength, which may underscore the risk for dementia. Finally, as the primarily of participants in the present study were white British, our findings may only generalizable to demographically similar cohorts.

## Conclusions

In summary, we found that both higher vitamin D and handgrip strength were associated with lower risk of incident dementia. The combination of ideal vitamin D and handgrip strength seemed to counteract the adverse effects of APOE e4 genotype on dementia by almost 50%. Our findings highlight that APOE e4 genotype carries may benefit from improving handgrip and avoiding vitamin D deficiency. Further clinical trials on handgrip strength and vitamin D interventions will be necessary to assess whether the observed associations are causal. Additionally, future studies could explore the underlying mechanisms by which vitamin D and handgrip strength may affect dementia risk, such as their effects on inflammation, oxidative stress, and neuroplasticity. Our findings, if confirmed by replications, may have implications for the development of dementia prevention strategies targeting the improvement of handgrip strength training and vitamin D supplementation, especially among individuals with APOE e4 carries.

## Supplementary Information

**Additional file 1.** **Table S1.** Joint exposure of handgrip strength and vitamin D with incidence of dementia in women and men according to age. **Table S2.** Joint exposure of handgrip strength and vitamin D with incidence of dementia in women and men according to education level. **Table S3.** Joint exposure of handgrip strength and vitamin D with incidence of dementia in women and men according to socioeconomic status. **Table S4.** Joint exposure of handgrip strength and vitamin D with incidence of dementia in women and men after excluding participants incident dementia or died during the first 3 years. **Table S5.** Joint exposure of handgrip strength and vitamin D with incidence of dementia in women and men after excluding participants incident major disease at baseline. **Table S6.** Association of vitamin D with incidence of dementia in women and men according to APOE e4 genotype. **Figure S1.** The relationship between grip strength and Vitamin D levels

## Data Availability

The data are available on application to the UK Biobank (www.ukbiobank.ac.uk/).
