# Supplementary material for: Ideal vitamin D and handgrip strength counteracts the risk effect of APOE genotype on dementia: a population-based longitudinal study
Source: J Transl Med. 2023 May 29;21:355. doi: 10.1186/s12967-023-04195-3 (PMC10226248; doi:10.1186/s12967-023-04195-3)
Supplement: Supplementary file 1 — Additional file 1. Table S1. Joint exposure of handgrip strength and vitamin D with incidence of dementia in women and men according to age. Table S2. Joint exposure of handgrip strength and vitamin D with incidence of dementia in women and men according to education level. Table S3. Joint exposure of handgrip strength and vitamin D with incidence of dementia in women and men according to socioeconomic status. Table S4. Joint exposure of handgrip strength and vitamin D with incidence of dementia in women and men after excluding participants incident dementia or died during the first 3 years. Table S5. Joint exposure of handgrip strength and vitamin D with incidence of dementia in women and men after excluding participants incident major disease at baseline. Table S6. Association of vitamin D with incidence of dementia in women and men according to APOE e4 genotype. Figure S1. The relationship between grip strength and Vitamin D levels [file 12967_2023_4195_MOESM1_ESM.docx]

**Additional Appendix:**

**Table S1.** Joint exposure of handgrip strength and vitamin D with incidence of dementia in women and men according to age

**Table S2.** Joint exposure of handgrip strength and vitamin D with incidence of dementia in women and men according to education level

**Table S3.** Joint exposure of handgrip strength and vitamin D with incidence of dementia in women and men according to socioeconomic status

**Table S4.** Joint exposure of handgrip strength and vitamin D with incidence of dementia in women and men after excluding participants incident dementia or died during the first 3 years

**Table S5.** Joint exposure of handgrip strength and vitamin D with incidence of dementia in women and men after excluding participants incident major disease at baseline

**Table S6.** Association of vitamin D with incidence of dementia in women and men according to APOE e4 genotype

**Figure S1.** The relationship between grip strength and Vitamin D levels

**Table S1.** Joint exposure of handgrip strength and vitamin D with incidence of dementia in women and men according to age

| Joint effect | | Women | |  | Men | |
| --- | --- | --- | --- | --- | --- | --- |
| Handgrip strength  /Vitamin D | APOE genotype | Age <65 | Age ≥65 |  | Age <65 | Age ≥65 |
| Low/low | APOE e4- | 1.00 (ref.) | 1.00 (ref.) |  | 1.00 (ref.) | 1.00 (ref.) |
| Intermediate | APOE e4- | 0.68 (0.50-0.94) | 0.78 (0.63-0.96) |  | 0.62 (0.47-0.81) | 0.63 (0.53-0.75) |
| High/high | APOE e4- | 0.45 (0.24-0.86) | 0.62 (0.39-0.99) |  | 0.38 (0.23-0.63) | 0.30 (0.19-0.46) |
| Low/low | APOE e4+ | 3.58 (2.47-5.20) | 4.58 (3.63-5.77) |  | 2.40 (1.72-3.34) | 2.72 (2.20-3.36) |
| Intermediate | APOE e4+ | 2.55 (1.87-3.49) | 2.84 (2.31-3.48) |  | 1.76 (1.35-2.29) | 1.88 (1.57-2.24) |
| High/high | APOE e4+ | 2.55 (1.56-4.16) | 2.27 (1.50-3.44) |  | 0.74 (0.42-1.32) | 1.57 (1.12-2.20) |

*Note:* Multivariate Cox regression models was adjusted for ethnicity, education level, socioeconomic status, alcohol consumption, smoking status, BMI, heart disease, stroke, hypertension, diabetes, depression, TC, HDL, and LDL.

**Table S2.** Joint exposure of handgrip strength and vitamin D with incidence of dementia in women and men according to education level

| Joint effect | | Women | |  | Men | |
| --- | --- | --- | --- | --- | --- | --- |
| Handgrip strength  /Vitamin D | APOE genotype | Low education  level | High education  level |  | Low education  level | High education  level |
| Low/low | APOE e4- | 1.00 (ref.) | 1.00 (ref.) |  | 1.00 (ref.) | 1.00 (ref.) |
| Intermediate | APOE e4- | 0.75 (0.48-1.18) | 0.75 (0.62-0.90) |  | 0.68 (0.49-0.93) | 0.61 (0.52-0.73) |
| High/high | APOE e4- | 0.47 (0.19-1.15) | 0.57 (0.38-0.87) |  | 0.31 (0.15-0.63) | 0.34 (0.23-0.49) |
| Low/low | APOE e4+ | 3.84 (2.29-6.45) | 4.36 (3.53-5.40) |  | 2.93 (2.00-4.28) | 2.54 (2.07-3.11) |
| Intermediate | APOE e4+ | 2.98 (1.93-4.60) | 2.71 (2.25-3.27) |  | 1.86 (1.35-2.56) | 1.84 (1.56-2.18) |
| High/high | APOE e4+ | 2.08 (0.96-4.49) | 2.48 (1.76-3.49) |  | 1.34 (0.75-2.40) | 1.21 (0.86-1.69) |

*Note:* Multivariate Cox regression models was adjusted for age, ethnicity, socioeconomic status, alcohol consumption, smoking status, BMI, heart disease, stroke, hypertension, diabetes, depression, TC, HDL, and LDL.

**Table S3.** Joint exposure of handgrip strength and vitamin D with incidence of dementia in women and men according to socioeconomic status

| Joint effect | | Women | | |  | Men | | |
| --- | --- | --- | --- | --- | --- | --- | --- | --- |
| Handgrip strength/Vitamin D | APOE genotype | Low TDI | Middle TDI | High TDI |  | Low TDI | Middle TDI | High TDI |
| Low/low | APOE e4- | 1.00 (ref.) | 1.00 (ref.) | 1.00 (ref.) |  | 1.00 (ref.) | 1.00 (ref.) | 1.00 (ref.) |
| Intermediate | APOE e4- | 0.61 (0.44-0.85) | 0.76 (0.55-1.06) | 0.85 (0.65-1.11) |  | 0.75 (0.55-1.04) | 0.70 (0.53-0.92) | 0.55 (0.44-0.68) |
| High/high | APOE e4- | 0.55 (0.30-1.01) | 0.45 (0.22-0.92) | 0.62 (0.32-1.20) |  | 0.47 (0.28-0.78) | 0.31 (0.17-0.57) | 0.26 (0.13-0.53) |
| Low/low | APOE e4+ | 4.39 (3.02-6.37) | 4.26 (2.92-6.22) | 4.29 (3.20-5.75) |  | 2.92 (1.98-4.32) | 3.16 (2.26-4.42) | 2.28 (1.77-2.93) |
| Intermediate | APOE e4+ | 2.23 (1.62-3.07) | 2.84 (2.06-3.91) | 3.14 (2.42-4.08) |  | 2.26 (1.65-3.09) | 2.09 (1.58-2.76) | 1.52 (1.22-1.90) |
| High/high | APOE e4+ | 1.59 (0.90-2.83) | 3.25 (1.96-5.41) | 2.42 (1.36-4.29) |  | 0.95 (0.53-1.68) | 1.85 (1.19-2.89) | 1.20 (0.68-2.14) |

*Note:* Multivariate Cox regression models was adjusted for age, ethnicity, education level, alcohol consumption, smoking status, BMI, heart disease, stroke, hypertension, diabetes, depression, TC, HDL, and LDL.

**Table S4.** Joint exposure of handgrip strength and vitamin D with incidence of dementia in women and men after excluding participants incident dementia or died during the first 3 years

| Joint effect | | Women |  | Men |
| --- | --- | --- | --- | --- |
| Handgrip strength/Vitamin D | APOE genotype | HR (95% CI) |  | HR (95% CI) |
| Low/low | APOE e4- | 1.00 (ref.) |  | 1.00 (ref.) |
| Intermediate | APOE e4- | 0.75 (0.63-0.90) |  | 0.62 (0.54-0.73) |
| High/high | APOE e4- | 0.53 (0.36-0.78) |  | 0.34 (0.25-0.48) |
| Low/low | APOE e4+ | 4.15 (3.40-5.08) |  | 2.65 (2.21-3.18) |
| Intermediate | APOE e4+ | 2.73 (2.30-3.25) |  | 1.86 (1.60-2.16) |
| High/high | APOE e4+ | 2.44 (1.79-3.34) |  | 1.24 (0.92-1.66) |

*Note:* Multivariate Cox regression models was adjusted for age, ethnicity, education level, socioeconomic status, alcohol consumption, smoking status, BMI, heart disease, stroke, hypertension, diabetes, depression, TC, HDL, and LDL.

**Table S5.** Joint exposure of handgrip strength and vitamin D with incidence of dementia in women and men after excluding participants incident major disease at baseline

| Joint effect | | Women |  | Men |
| --- | --- | --- | --- | --- |
| Handgrip strength/Vitamin D | APOE genotype |  |  |  |
| Low/low | APOE e4- | 1.00 (ref.) |  | 1.00 (ref.) |
| Intermediate | APOE e4- | 0.69 (0.57-0.85) |  | 0.61 (0.50-0.75) |
| High/high | APOE e4- | 0.46 (0.29-0.71) |  | 0.27 (0.18-0.42) |
| Low/low | APOE e4+ | 4.47 (3.55-5.62) |  | 3.12 (2.46-3.96) |
| Intermediate | APOE e4+ | 2.85 (2.34-3.48) |  | 2.01 (1.65-2.45) |
| High/high | APOE e4+ | 2.64 (1.89-3.68) |  | 1.41 (1.01-1.98) |

*Note:* Multivariate Cox regression models was adjusted for age, ethnicity, education level, socioeconomic status, alcohol consumption, smoking status, BMI, heart disease, stroke, hypertension, diabetes, depression, TC, HDL, and LDL.

**Table S6. Association of vitamin D with incidence of dementia in women and men according to APOE e4 genotype**

|  | Women |  |  | Men |  |
| --- | --- | --- | --- | --- | --- |
| Vitamin D | APOE e4- | APOE e4+ |  | APOE e4- | APOE e4+ |
| Low | 1.00 (ref.) | 1.00 (ref.) |  | 1.00 (ref.) | 1.00 (ref.) |
| Middle | 1.01 (0.84-1.21) | 0.77 (0.65-0.90) |  | 0.76 (0.64-0.89) | 0.84 (0.72-0.98) |
| High | 0.91 (0.76-1.08) | 0.73 (0.63-0.85) |  | 0.65 (0.56-0.75) | 0.80 (0.69-0.92) |

^a^Multivariate Cox regression models were adjusted for age, ethnicity, education level, socioeconomic status, alcohol consumption, smoking status, BMI, heart disease, stroke, hypertension, diabetes, depression, TC, HDL, LDL, and grip strength.

**
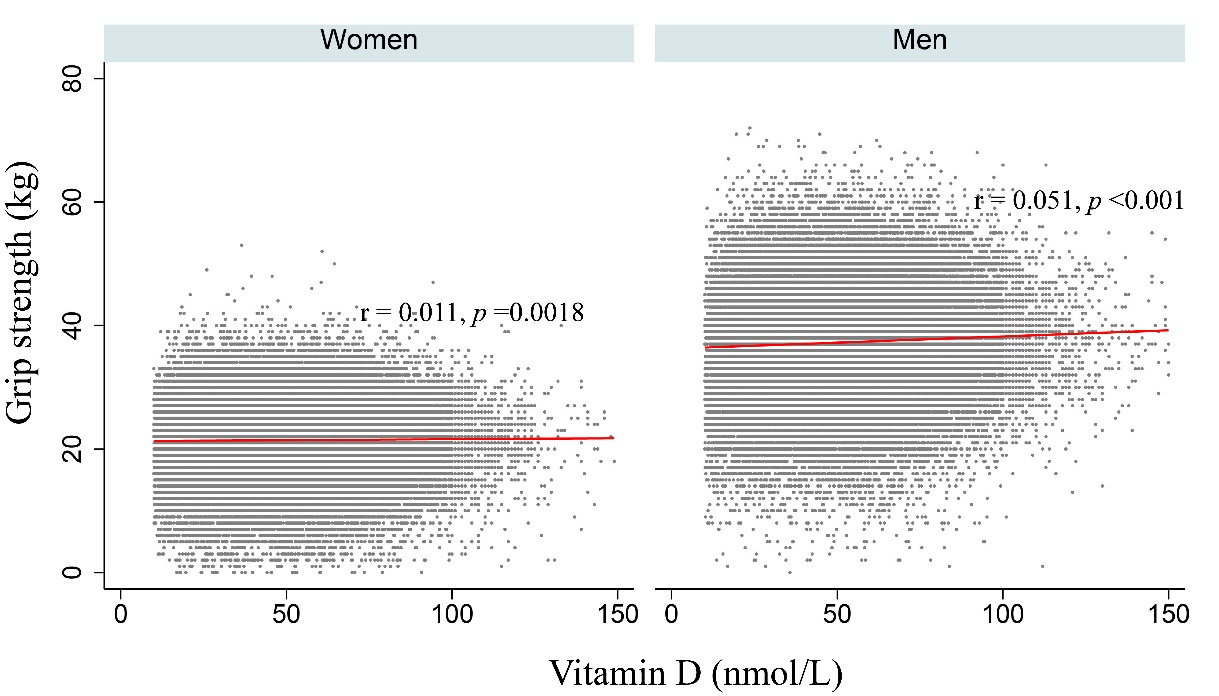
**

**Figure S1.** The relationship between grip strength and Vitamin D levels
